# An Adult Case of Crouzon Syndrome: Diagnostic Features and Treatment Modalities

**DOI:** 10.7759/cureus.59605

**Published:** 2024-05-03

**Authors:** Farhad Sobouti, Sepideh Dadgar, Negareh Salehabadi, Anahita Lotfizadeh, Ali Mazandarani, Mehdi Aryana

**Affiliations:** 1 Department of Orthodontics, Faculty of Dentistry, Mazandaran University of Medical Sciences, Sari, IRN; 2 Student Research Committee, Faculty of Dentistry, Mazandaran University of Medical Sciences, Sari, IRN

**Keywords:** orthosurgery, orthodontics, genetic diseases, crouzon syndrome, craniosynostoses

## Abstract

Craniosynostosis syndromes are birth defects characterized by the premature fusion of one or more cranial sutures before the completion of brain growth and development. Crouzon syndrome (CS) is the most common craniosynostosis condition. The CS manifestations result from the early fusion of superior and posterior sutures of the maxilla along the orbital wall and affect the cranial vault, base, orbital, and maxillary regions. This report presents a rare case of a 25-year-old male CS patient referred for orthodontic treatment with the chief complaint of severe irregularities in the arrangement of teeth and abnormal facial appearance. In this report, the clinical, cephalometric features, and initial orthodontic management of this patient are discussed as part of multidisciplinary management.

## Introduction

Craniosynostosis syndromes are birth defects characterized by the premature fusion of one or more cranial sutures before the completion of brain growth and development. These syndromes cause restricted development of the skull, brain, face, and central nervous system [1]. The most common craniosynostosis syndromes include Crouzon, Apert, Pfeiffer, Carpenter, Saethre-Chotzen, and Jackson-Weiss syndromes. Crouzon syndrome (CS), the most common craniosynostosis condition, is a rare genetic disorder with a global prevalence of 16 per million births [2]. It is an autosomal dominant condition linked to numerous fibroblast growth factor receptor 2 (FGFR2) mutations. FGFR2 has a signaling function in cranial sutures and plays a crucial role in limb embryonic development [3]. The first cases of CS were diagnosed in 1912, after the identification of a triad of calvarial deformities with craniofacial dysostosis, exophthalmos, and facial anomalies in a mother and her son [4].

CS manifestations result from the early fusion of superior and posterior sutures of the maxilla along the orbital wall, which can affect the cranial vault, base, orbital, and maxillary regions [5]. The sutures’ growth potential becomes restricted after the fusion. The order and extent of fusions determine the type of deformity and the associated inability [6]. Common manifestations of CS are coronal craniosynostosis with other cranial suture fusion, brachycephaly, hypertelorism, frontal bossing, strabismus, orbital proptosis, mandibular prognathism, and maxillary hypoplasia. These characteristics either become more prominent or regress through time. Hearing loss is prevalent (55%), and C2 and C3 spinal fusion occur at a rate of 30% [7]. Progressive hydrocephalus (30%), tonsillar herniation, and sacrococcygeal tail are some of the other symptoms. Extremity and mental capacity are usually normal in these patients. However, early closure of the cranial suture lines can cause mental retardation if it inhibits brain development due to persistently elevated intracranial pressure [8]. Here, we present a rare case of CS referred for orthodontic treatment and a review of the related literature.

This article was previously posted to the Authorea preprint server on December 12, 2022 (doi: 10.22541/au.167084122.29914012/v1).

## Case presentation

A 25-year-old male patient was referred to a private orthodontic clinic in Sari, Iran, with the chief complaint of severe irregularities in teeth arrangement and abnormal facial appearance. His dentoskeletal abnormality caused speaking and chewing difficulties. He was the third child in a family of six children. Neither the patient nor his parents had attended university. According to his family history, one of his cousins had CS. The patient hadn’t visited a dentist until this age due to his parents' lack of attention. He was psychologically abused due to his poor facial proportion, resulting in employment problems and social isolation. The patient had hypertension, which was related to his weight and serum low-density lipoprotein (LDL) levels; as a result, he needed two different types of antihypertensive medications to keep his blood pressure under control.

Clinical examination

**Figure 1:**
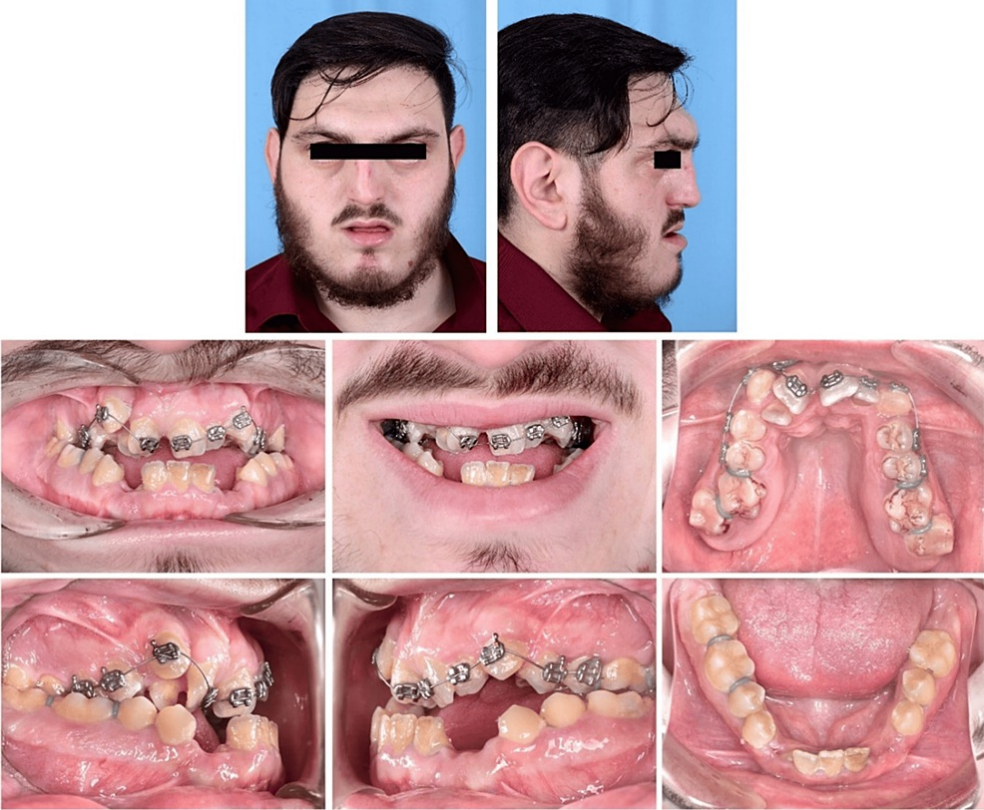
Patient’s extraoral and intraoral photographs on the first day of orthodontic bonding

Facial examination revealed a severely concave profile with a prominent superciliary arch and an obvious midface deformity. The patient was under the care of an ophthalmologist due to moderate hypertelorism and ocular proptosis. His nasal bridge and tip were deviated. The patient’s mental development and function were normal, but he was under the care of a neurologist due to recurrent headaches caused by increased intracranial pressure. According to the otolaryngologist's assessment, he had severe sleep apnea with an apnea-hypopnea index of greater than 15 and a respiratory disturbances index score of 15 due to a retruded nasomaxillary complex. His face was symmetric from an orthodontic standpoint, with a proportionate vertical 1/5 and a decreased lower midfacial height (Figure 1).

Intraoral examination

Gingival inflammation and poor oral hygiene were identified. The upper labial frenum was highly attached and fibrotic. Due to significant transverse maxillary deficiency, there was severe crowding in the maxillary arch (12 mm), bilateral posterior crossbite, and a deep palatal vault. Molar and canine relationships were class III on both sides, with a 2 mm anterior open bite and a -4 mm overjet. The upper central incisors and right first molar had severe rotation. Both canines and third molars, the left lateral incisor, and the left second molar were missing from the mandibular dental arch (Figure 1).

Radiographic examination

Cephalometric Radiography Findings

**Figure 2:**
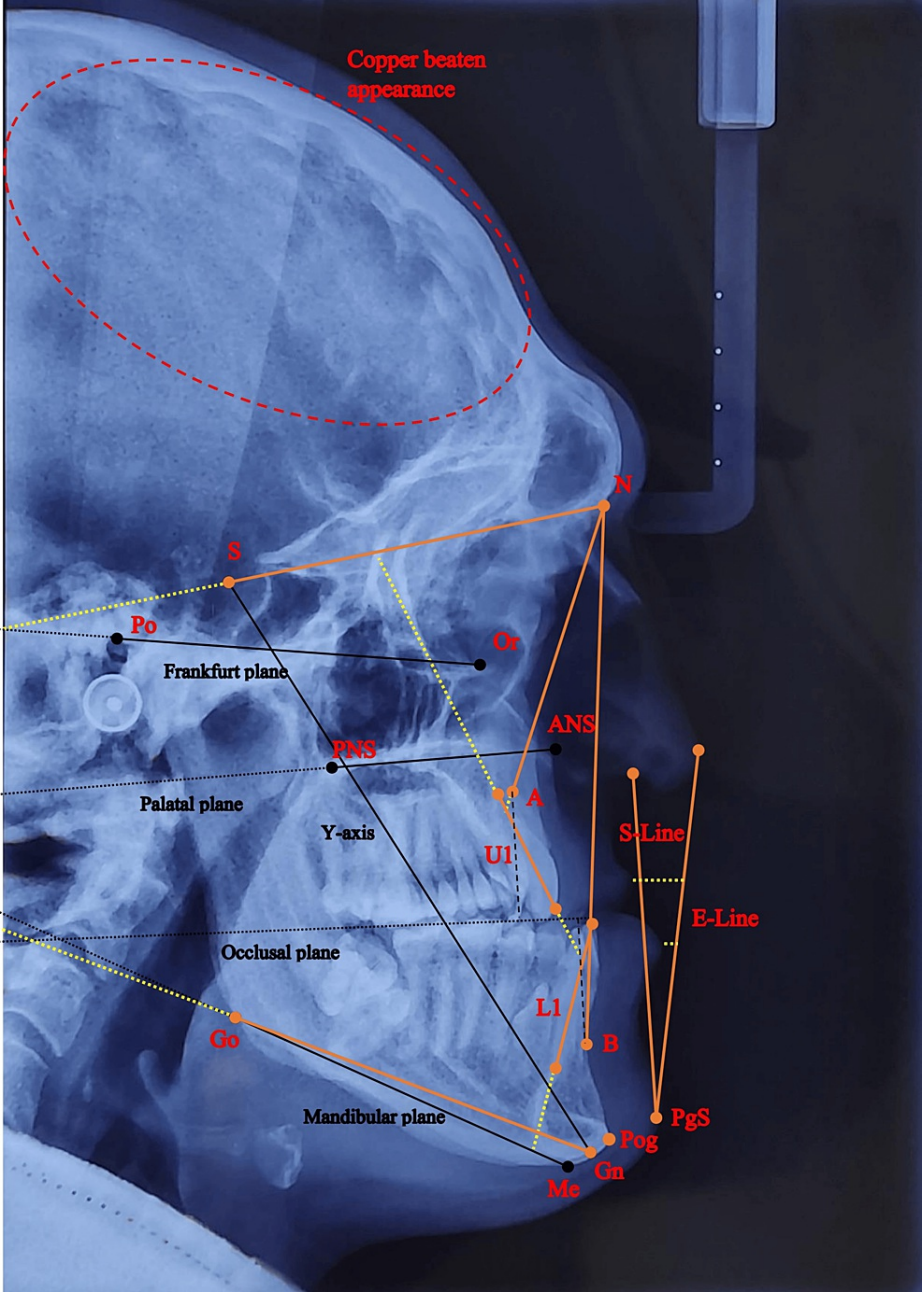
Patient's pretreatment lateral cephalogram A: subspinale, ANS: anterior nasal spine, B: supramentale, Gn: gnathion, Go: gonion, L1: lower (mandibular) central incisor, Me: menton, N: nasion, Or: lowest point on the border of orbital fossa, PgS: soft tissue pogonion, PNS: posterior nasal spine, Po: porion, Pog: pogonion, S: sella, U1: upper (maxillary) central incisor

Cephalometric analysis was performed via a Persian artificial intelligence-based automatic tracing software, Orthoyar (Mazandaran University of Medical Sciences, Sari, Iran) [9]. The analysis demonstrated that the maxilla was severely retruded, although the lower jaw position was reasonably normal compared to the facial skeleton (Figure 2 and Table 1). Upper and lower incisors were nearly retroclined, and all sagittal measures indicated a severe skeletal Class III malocclusion. The most noticeable characteristic of the patient’s cranium was copper beaten appearance, which are prominent convolutional markings seen in multiple bones of the skull. These markings are frequent in craniosynostosis patients and can be a sign of elevated intracranial pressure in these patients [10].​​​​​

**Table 1:** Cephalometric measurements A: subspinale, ANS: anterior nasal spine, B: supramentale, Gn: gnathion, Go: gonion, L1: lower (mandibular) central incisor, Me: menton, N: nasion, N perpendicular: the straight line from nasion perpendicular to horizon, Or: lowest point on the border of orbital fossa, PgS: soft tissue pogonion, PNS: posterior nasal spine, Po: porion, Pog: pogonion, S: sella, U1: upper (maxillary) central incisor, Basal angle: palatal to mandibular plane angle, Interincisal angle: U1 to L1 angle, IMPA angle: L1-GoGn angle, FMA angle: Frankfort-GoGn angle

Landmark	Value
SNA angle	61.1º
SNB angle	76.7º
ANB angle	-15.6º
Basal angle	28.5º
SN-GoGn angle	32.3º
Upper lip to S-line distance	-1.9 mm
Interincisal angle	139.4º
A point to N _perpendicular _distance	-12.4 mm
U1 to SN angle	74.7º
IMPA angle	83.2º
FMA angle	16.7º
U1 to Palatal plane angle	68º
Upper lip to E-line distance	-7 mm
Lower lip to E-line distance	-3 mm
Y-axis to SN angle	69.3º
Wits appraisal	-9 mm

Panoramic Radiography Findings

**Figure 3:**
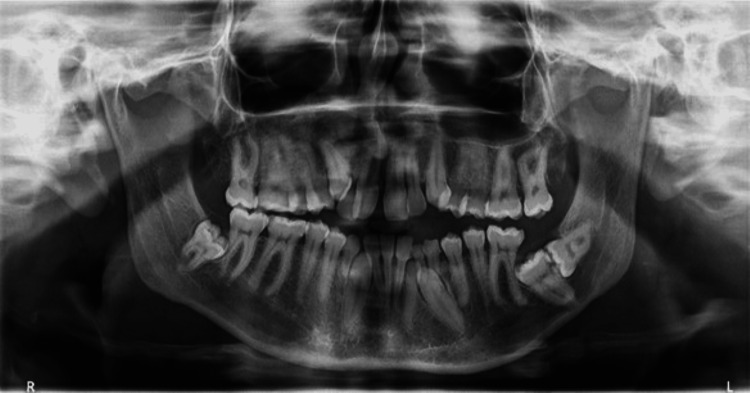
Patient's pretreatment panoramic radiography

The patient's upper third molars were missing congenitally. In the mandible, deciduous canines and left lateral incisor, and permanent canines, left lateral incisor, left second molar, and both third molars were impacted. Roots were longer than normal in both jaws, and condylar necks were nearly extended. Permanent teeth were free of cavities. The mandibular canals were quite wide and visible in the panoramic view. A deep and visible sigmoid notch was detectable on both sides. There was also a deviated nasal septum and a deformed distorted space (Figure 3).

*Cone-Beam CT* (*CBCT) Findings*

**Figure 4:**
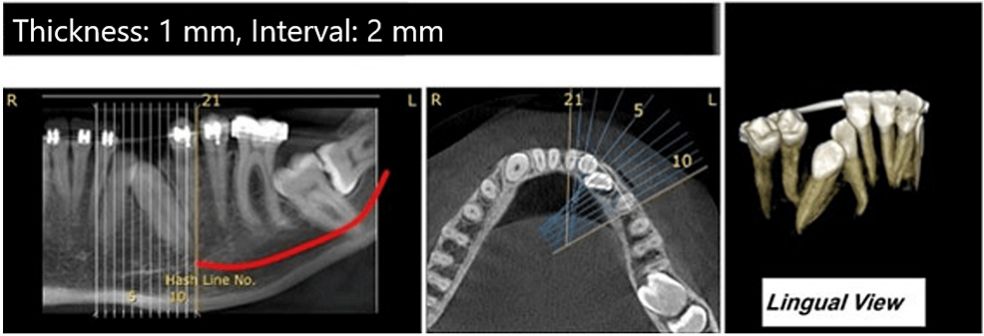
Pretreatment cone-beam CT image of anterior mandibular teeth

Three-dimensional and axial views of CBCT (NewTom 9000; Quantitative Radiology, Verona, Italy) were provided to determine the precise position and angulation of the impacted mandibular permanent incisors and canines. According to CBCT slide analysis, better positions of anterior impacted teeth made them better candidates for orthodontic eruption paired with surgical exposure (Figure 4). However, the crown of the left second molar and the distal root of the first molar had a close relationship in the posterior region of the mandible (Figure 3). On the right side, the third molar was severely angulated and deeply impacted, making the surgery more challenging (Figure 3).

Clinical diagnosis of CS

The CS diagnosis was obtained after clinical and radiological investigations. The signs of cranial deformities, midface hypoplasia, three-dimensional (3D) maxillary deficiency (vertical, transverse, and anteroposterior), mandibular prognathism, hypertelorism, proptosis, short upper lip, severe crowding of teeth, and copper beaten appearance of the skull all led to the diagnosis of CS in the patient.

Orthodontic diagnosis

According to the clinical and paraclinical databases, the patient was diagnosed with (i) Concave profile, increased lower anterior face height (LAFH), retruded midface, lip incompetence, short upper lip, reduced tooth showing while smiling, severe crowding in the upper jaw, wide buccal corridor; (ii) Class III malocclusion, reversed overjet, several impacted teeth in anterior and posterior segments of the mandible, severe rotation in maxillary incisors, over-retained primary teeth in the mandibular arch, deep palatal vault, severe posterior crossbite; (iii) Skeletal Class III due to severe maxillary deficiency; (iv) Severe maxillary transverse deficiency; and (v) Mild excess in the vertical dimension of the maxillomandibular complex

Treatment objectives

Several steps are recommended to comprehensively treat the patient’s dentofacial anomalies. Orthognathic treatment is an essential part of the treatment plan. However, due to multiple impacted teeth and extreme maxillary constriction, treatment approaches may be more challenging than in common orthognathic patients. In adults, such as our patient, directed eruption of impacted teeth to the lower dental arch can be risky due to the likelihood of ankylosis during tooth movement. Also, severe maxillary constriction makes it difficult to develop proper arch coordination between the maxilla and mandible. Since transverse dimensions harmony in such cases is critical for achieving stable and functional results, selecting the most beneficial surgical approach to expand the maxilla is crucial. Another challenge will be the amount of reverse overjet, which defines the amount of forward displacement of the maxilla based on the 3D surgical prediction after initial alignment and leveling. As a result, although the risk of relapse is diminished in class III patients with maxillary advancement surgery, such severe skeletal discrepancy may be susceptible to relapse. Hence, some surgeons and orthodontists prefer advancing the maxilla by distraction osteogenesis (DO) technique, which can yield more stable results [11].

Treatment plan

The initial step of the treatment planned was the extraction of lower third molars. Then, surgically-assisted rapid palatal expansion (SARPE) could be carried out via Le Fort I osteotomy to expand the hypoplastic maxilla, and tooth-borne fixed banded rigid expanders could be utilized for premolars and molars to provide enough space for tooth alignment and skeletal expansion in the maxilla. Next, preadjusted 0.022-inch slot MBT brackets (American Orthodontics, Sheboygan, Wisconsin, United States) will be bonded and a wire sequence of 0.014-, 0.016-, and 0.018-inch austenite nickel-titanium (A-NiTi), 0.017×0.025-inch and 0.019×0.025-inch martensite (M)-NiTi, and 0.018-inch stainless steel (American Orthodontics) will be used for alignment and leveling in both arches.

Once the 0.018-inch stainless steel wire has been inserted and enough space has been gained, all impacted teeth will be surgically exposed in two sessions using the periodontal apically positioned flap technique, which will preserve enough keratinized tissue in the surgical site. Then, a 10×1.8 mm tapered miniscrew (AbsoAnchor; Dentos Inc., Daegu, Korea) should be used as a temporary anchorage device in the lower left quadrant's retromolar area to upright the impacted second molar. The impacted teeth will be repositioned through the bonding of orthodontic attachments to the teeth surface, utilizing the elastic power chains.

Following a consultation with an oral and maxillofacial surgeon and a 3D virtual surgical plan assessment, the next step should be maxillary advancement using an extraoral device for DO. Then, surgical wires and hooks (0.021×0.025-inch stainless steel; American Orthodontics) will be inserted in both arches. Following a minimum of two weeks of stabilization using heavy elastics to preserve the surgical site, the surgical 0.021×0.025-inch stainless steel wires will be replaced with light 0.016-inch stainless steel wires (American Orthodontics). Along with using inter-arch orthodontic elastics, an optimal functional occlusion could be achieved. Next, 0.016-inch stainless steel wires (American Orthodontics) will be used for orthodontic finishing and detailing. After debonding all attachments, results will be stabilized with fixed retainers in both arches and maintenance instructions. A removable Hawley retainer will also be placed in the maxilla.

Alternative treatment plan

After bonding with preadjusted 0.022-inch slot MBT bracket system (American Orthodontics) in both arches, alignment and leveling will be done with the wire sequence of 0.014-, 0.016-, and 0.018-inch ANiTi, 0.017×0.025-inch and 0.019×0.025-inch MNiTi, and 0.018-inch stainless steel wires (American Orthodontics). While using the 0.018-inch stainless steel wire, surgical exposure and orthodontic traction of the impacted teeth will be carried out. Miniscrew and elastomeric traction module will be used to upright the lower left second molar. After 3D virtual presurgical assessment, maxillary segmental osteotomy (two pieces) will be carried out for maxillary skeletal expansion and Le Fort III osteotomy for maxillary advancement.

If needed, mandibular bilateral sagittal split osteotomy could be performed for mandibular setback. The post-expansion outcomes will be maintained by using a soldered transpalatal arch. The subsequent phase of orthodontic treatment will begin two weeks after surgery. The surgical wires will be removed and light wires (0.016-inch stainless steel; American Orthodontics) will be inserted together with inter-arch elastics, and the treatment will endure for four months. Next, 0.018-inch stainless steel wire will be used for finishing and detailing. Finally, a fixed lingual retainer will be bonded in the lower arch and a removable Hawley appliance will be used in the maxilla.

## Discussion

Craniofacial anomalies are frequently present at birth and might get worse over time. CS patients should receive orthodontic evaluations at a young age. Early treatment allows obtaining higher possibilities to correct dental issues and facial asymmetry and prevent further complications such as increased intracranial pressure, respiratory distress, and optic atrophy [7]. Surgical extraction of several permanent teeth could reduce the crowding in the dental arch and the use of orthodontic appliances could expand the maxilla [12]. In this case, addressing the issue of crowding could be time-consuming due to several teeth rotations and severe mal-alignment. In addition, alignment and leveling of multiple impacted permanent teeth increase the difficulty of the treatment. Therefore, the use of rapid tooth movement techniques such as photobiomodulation therapy and micro-osteoperforation (corticotomy-assisted tooth movement) could accelerate the movement and shorten the alignment period [13]. Also, laser surgery could be utilized to recontour the gingival margin of the impacted teeth [14].

Surgical management of CS patients is suggested in the literature to reduce facial and dental problems. This includes a series of multistage procedures such as craniectomy, posterior vault expansion, DO, and Le Fort III advancements [15]. Skull reshaping, midfacial advancement, jaw surgery, and orthopedic and orthodontic approaches are all beneficial measures that could be used in CS patients [3,12,16,17]. Since our patient presented after cessation of growth, no growth modification therapy could be administered.

Mohammadi et al. have listed the various surgical procedures that are typically recommended at this point as follows: (i) subcranial monobloc advancement osteotomy for the correction of deficiencies in the infraorbital, supraorbital, and nasomaxillary areas; (ii) Le Fort III extracranial osteotomy for the correction of defects located in the middle third of the face in patients with normal frontal areas; (iii) modified Le Fort III osteotomy for indicating where the nasomaxillary complex is not involved, and only the midface needs correction; and (iv) Le Fort II osteotomy for indicating where the orbital and zygomatic areas are minimally involved compared to the nasomaxillary areas [18].

Due to the greater complications of the monobloc Le Fort III advancements [19], the Le Fort III osteotomy with DO for advancement might be the preferred treatment in our case. Increased stability and range of displacement in severe deficiencies without the necessity for internal rigid fixation are two advantages of DO. Furthermore, the requirement for bone transplants, as well as the related morbidities and expenses, can be avoided [20]. Since the anteroposterior and transverse discrepancies, in this case, were severe, DO surgery could be the only option to obtain greater stability since it allows for the gradual expansion and histogenesis of the soft tissues as well as skeletal augmentation [11].

## Conclusions

CS was diagnosed in the 25-year-old male patient based on the clinical and radiographic findings of hypertelorism, ocular proptosis, increased intracranial pressure, severe sleep apnea, midface hypoplasia, 3D maxillary deficiency, mandibular prognathism, short upper lip, severe crowding of teeth, and copper beaten appearance of the skull. The treatment plan included extraction of impacted lower third molars, maxillary skeletal expansion, alignment and leveling of the teeth in both jaws, surgical exposure and orthodontic traction of the impacted permanent teeth, surgical maxillary advancement, post-surgical orthodontic tooth movement to provide ideal functional occlusion, and orthodontic finishing and detailing.
